# Perspectives of patients undergoing neoadjuvant chemotherapy for breast cancer during the COVID‐19 pandemic

**DOI:** 10.1002/cnr2.1882

**Published:** 2023-08-16

**Authors:** Alice A. Gaughan, Sarah R. MacEwan, Laura J. Rush, Margaret E. Gatti‐Mays, Ashley C. Pariser, Ann Scheck McAlearney

**Affiliations:** ^1^ The Center for the Advancement of Team Science, Analytics, and Systems Thinking in Health Services and Implementation Science Research (CATALYST), College of Medicine The Ohio State University Columbus OH USA; ^2^ Division of General Internal Medicine College of Medicine, The Ohio State University Columbus OH USA; ^3^ Pelotonia Institute for Immuno‐Oncology, Division of Medical Oncology The Ohio State University Comprehensive Cancer Center – The James Columbus Ohio USA; ^4^ Department of Family and Community Medicine College of Medicine, The Ohio State University Columbus OH USA; ^5^ Department of Biomedical Informatics College of Medicine, The Ohio State University Columbus OH USA

**Keywords:** breast cancer, cancer screening, COVID‐19, mammogram, vaccination

## Abstract

**Background:**

The COVID‐19 pandemic resulted in a lapse in routine health care and cancer screenings for many individuals. This study sought to improve our understanding of the impact of the COVID‐19 pandemic on women being treated for breast cancer, both in general, and specifically related to their diagnosis.

**Methods:**

Semi‐structured interviews were conducted between August 2021 and February 2022 with women who were receiving neoadjuvant chemotherapy for early‐stage breast cancer at the Stefanie Spielman Comprehensive Breast Center in Columbus, Ohio. Interviews were recorded and transcribed verbatim. Transcripts were coded using deductive dominant thematic analysis and inductive coding that allowed for categorization of data as well as identification of emergent themes.

**Results:**

Data collected from our 19 interviews revealed that the COVID‐19 pandemic posed important challenges for breast cancer patients including fear of COVID‐19 infection and feelings of isolation. Most interviewees noted they had been vaccinated against COVID‐19 because of a desire to protect themselves and others from getting sick. Some women also expressed concerns about having delayed their screening mammograms due to the pandemic. Several patients described unexpected positive aspects of the pandemic such as being able to spend more time with family and having the ability to continue working because of the option to work from home during their cancer treatment.

**Conclusions:**

Our findings provide important insight about the impact of COVID‐19 on breast cancer patients. We highlight the positives that have been reported because of the pandemic, as well as the need to address delayed breast cancer screening.

## 1 INTRODUCTION

The World Health Organization declared a COVID‐19 pandemic in early March 2020,^1^ and the United States (U.S.) initiated a major shutdown shortly thereafter.^2^, ^3^ At that time, the U.S. health care system underwent a rapid transition. Hospitals quickly became overwhelmed by patients with COVID‐19, and resources were directed toward care for critically ill people. Elective procedures were halted, outpatient care was severely restricted, and routine imaging services were canceled.^4^, ^5^, ^6^ Among the many downstream consequences associated with these reductions in non‐emergent health care was a marked decrease in cancer screening, including routine screening mammograms.^7^

Screening mammography for breast cancer is recommended starting at age 45 for women who are at average risk of disease.^8^ When breast cancer is diagnosed early, with only localized disease, there is a 99% 5‐year survival rate versus a 29% 5‐year survival rate when distant metastases are present.^9^ Annually, 39 million women get screening mammograms in the U.S.,^10^ and although breast cancer remains the most common type of cancer diagnosed in women (31% of all cancers), it accounts for only 15% of cancer‐related deaths in women.^11^ Unfortunately, studies have demonstrated a massive reduction in the number of screening mammograms performed during the pandemic, with almost 90% fewer mammograms performed in early 2020 versus the same period in 2019.^12^, ^13^ The reduction in early detection of breast cancer and the delayed diagnosis means that more women will be diagnosed at an advanced stage of disease, which is less amenable to curative treatment.^14^, ^15^ Even with the availability of vaccinations against COVID‐19, the easing of public health restrictions, and return of availability of routine screening procedures, some women may not feel comfortable going out in public and entering health care facilities, further exacerbating the delay in timely diagnosis.

While evidence of the impact of the pandemic on breast cancer incidence rates and care have continued to grow, less attention has been paid to the experiences of patients as they have navigated challenges imposed by the pandemic while undergoing treatment for breast cancer. Several studies have reported experiences of breast cancer patients during the early waves of the pandemic^16^, ^17^, ^18^ or experiences of breast cancer survivors.^18^, ^19^, ^20^ Yet few have evaluated the perspectives of breast cancer patients undergoing treatment as the pandemic evolved. The landscape of public health measures and health care accessibility changed throughout the pandemic, including changes in reaction to the emergence of COVID variants and changes set in motion by the availability of COVID‐19 vaccines. As such, we expect patients to have perspectives that may be unique to points in time across the pandemic period.

As part of a larger study examining the effects of the COVID‐19 pandemic, we interviewed women undergoing neoadjuvant treatment for early‐stage breast cancer to understand how the COVID‐19 pandemic had impacted their lives and their cancer treatment journeys. These interviews captured patient perspectives in August 2021 to February 2022, a period that encompassed the delta and omicron variant waves and during which COVID vaccines were widely available in the United States. We aimed to learn about women's decisions about screening and treatment, as well as any unexpected sequelae that these women identified during this trying time.

## 2 MATERIALS AND METHODS

### 2.1 Study setting and population

We conducted a qualitative study comprised of interviews with women who were being treated for early‐stage breast cancer at the Stefanie Spielman Comprehensive Breast Center within The Ohio State University Comprehensive Cancer Center (OSUCCC) in Columbus, Ohio. Interviews were held between August 2021 and February 2022 and aimed to understand how breast cancer patients were impacted by the COVID‐19 global pandemic. During the time of the study, COVID‐19 vaccinations were readily available. This study was approved by The Ohio State University's Institutional Review Board.

### 2.2 Study design, data collection, and interview procedures

Breast cancer patients were recruited to participate in study interviews with the help of their care team, receiving a recruitment flyer from their care team. Interested patients then received an email from study team members with additional information about the study and how to participate. Patients who were at least 18 years old, were receiving neoadjuvant chemotherapy for early‐stage breast cancer, and were fluent in English were eligible to participate in the study. Interested patients contacted the study team to be scheduled for interviews. We conducted one‐on‐one phone interviews using a semi‐structured interview guide (see Appendix A) with individuals who reached out to participate.

All interviewees provided verbal informed consent prior to participating in the study. Interviews were conducted by phone using a semi‐structured interview guide that asked questions about COVID‐19 vaccination, risk, testing, guidance, as well as the impact of COVID‐19 on experiences with health care and mental health. Interviews lasted an average of 26 min (range = 12–46 min) with interview length determined by the responses of the interviewee. The interviews were audio‐recorded, transcribed verbatim, and de‐identified for analysis.

### 2.3 Data analysis

We used deductive dominant thematic analysis^21^, ^22^ to code and analyze interview transcripts. This allowed for categorization of data based on general themes derived from the interview guide, as well as identification of emergent themes. One study team member performed preliminary coding. At least two study team members then performed secondary coding, meeting frequently to ensure consistent application of the codes as well as identification and application of any emergent codes across all transcripts. This constant comparative approach allowed for comparison of themes across interviews and characterization of themes around the impact of the COVID‐19 pandemic on breast cancer patients. This approach also ensured that we reached saturation in our data collection with respect to the themes we present. ATLAS.ti software (ATLAS.ti Scientific Software Development: Berlin, Germany) was used to support our qualitative coding and analysis process.

## 3 RESULTS

### 3.1 Characteristics of study participants

As shown in Table 1, the interviewees included 19 breast cancer patients with an average age of 56 years (range = 29–69 years), with most between 60 and 69 years of age (10/19; 53%). During interviews, participants were asked about their COVID‐19 vaccination status and most indicated they had received at least one dose of a vaccine (14/19; 74%).

**TABLE 1:** Demographics of breast cancer patients interviewed.

Age (years)	Overall	Vaccinated
	*N* = 19	*N* = 14 (74%)
	*n* (%)	*n* (%)
20–29	1 (5)	0 (0)
30–39	2 (11)	2 (100)
40–49	3 (16)	1 (33)
50–59	3 (16)	2 (66)
60–69	10 (53)	9 (90)

### 3.2 Perspectives on COVID‐19 vaccination

We found that most interviewees were consistent in their perspectives that COVID‐19 vaccination protected them and others from getting sick, although some had concerns about vaccine safety. Perspectives of vaccinated and unvaccinated participants are presented below, with additional representative quotations provided in Table 2.

**TABLE 2:** Perspectives on COVID‐19 vaccination.

Theme	Perspectives of breast cancer patients
Accepting vaccination to protect themselves and others from getting sick	*I just didn't want to get sick. I didn't want to get it [COVID‐19], so I took the vaccine. (vaccinated)* *It was a very easy decision for me because I understand the science and I understand how, you know, FDA processes, and how such things happen, and I understood mRNA technology. So, for me, it was a very easy decision and sort of a no‐brainer. (vaccinated)*
Concerns about vaccine safety	*I feel like that they hadn't had, really had a chance to really test it to see what the positive sides or the negative sides of it could, might end up being. (vaccinated)* *I don't like the way it was rushed to market. It has nothing to do with politics. I don't think we're being told the truth about the death totals of COVID, and I don't believe that we're being told the truth about the complications that people have suffered from COVID. I think that from my personal experience of people that I know who had the shot, I'm not going near it. I just can't do it. (unvaccinated)*
Delaying vaccination due to medical care/health status	*They [doctors] wanted me to wait till I got all my testing done and then I'm going to go in next week and get my booster. (vaccinated)* *It had just got approved while I was pregnant, so I just wanted to wait until after I gave birth. During pregnancy, I got diagnosed so everything kind of took over. (unvaccinated)*

All vaccinated participants explained that the reason they received a COVID‐19 vaccine was to protect themselves and others from getting sick. As one vaccinated interviewee shared, “*I thought, both for myself personally, it was a good way to protect me and my family. I've got kids at home. I wanted to prevent them from possibly getting sick since they didn't have access to the vaccine, and they were going to school and such. But also, I just felt like it was community‐wise the right thing to do as a member of the community to help stop the spread*.” Another interviewee noted initially having concerns about vaccine safety by explaining: “*When I found out I had cancer, my oncologist highly recommended me getting it. I had concerns about the safety because I actually live in a community where a lot of people are not still vaccinated. And I guess you just hear other people talk about things they have read or things they have heard. And so, I did look into it myself before I got it, and decided it was the better option*.”

Unvaccinated interviewees were divided in their perspectives about the safety of COVID‐19 vaccination. One unvaccinated participant noted wanting more information about the vaccine before getting vaccinated by sharing, “*Well, if there was more research and the FDA approved more vaccines, I want to see more research on it*.” In contrast, other unvaccinated interviewees explained that delays in getting vaccinated were due to medical reasons. For example, an unvaccinated participant shared, “*I kind of just put it on hold, knowing I was going to have chemo. And I did speak with both my oncologist at Ohio State and at Cleveland Clinic about it. So, no, I'm not opposed to it. I just was holding off until I am through with chemo*.”

### 3.3 Impact of COVID‐19 pandemic on health care

Breast cancer patients we interviewed emphasized that the COVID‐19 pandemic had had a major impact on their health care. One participant noted that the COVID‐19 pandemic restricted activity altogether, sharing, “*I think I just wasn't going anywhere. It wasn't necessarily that I was putting off doing medical stuff because I was afraid to do medical stuff, I think it was more along the lines of I just wasn't going anywhere*.” However, many breast cancer patients we interviewed specifically noted the pandemic delayed their screening mammogram in the year prior to their diagnosis. For example, one interviewee shared, “*I probably would have got my mammogram sooner had it not been for COVID. I would have. I was supposed to come in for my mammogram and, as you know, we shut down everything. COVID shut things down for a full year*.” Another breast cancer patient similarly shared, “*I did put off my mammogram a year. My doctor had told me that I needed to go get one and I was like yeah, I'll go get it. And then, when I went in for it, August of 2020, she's like, you still haven't gotten it, you need to go get it. And I was like, okay. And I went and got it and that's, I mean, good thing I did because that's when they found the cancer in there*.”

Delays of other medical procedures, testing, and appointments due to the COVID‐19 pandemic were also reported by breast cancer patients. One interviewee explained, “*They delayed my stuff [medical procedure] because they didn't have staff because of COVID. They had to delay putting my port in, so I had to get my first set of chemo without my port in because they didn't have the staffing*.” Another interviewee explained delaying other procedures stating, “*I've delayed other things too because of COVID. After being in the hospital, they suggested that I have a very invasive test like a scope. I was like no way. I'm not doing that. I'll wait till after maybe, you know, see how long COVID is here*.” A third participant expressed concern about delays in cancer‐related testing. She shared, “*I will tell you what COVID has delayed substantially is the genetic assessment [for breast cancer], especially in young women. … Genetics was considered a non‐essential service. And … so they have a big backlog*.” Additional representative quotations showing the perspectives about the impact of the COVID‐19 pandemic on health care are listed in Table 3.

**TABLE 3:** Perspectives on the COVID‐19 pandemic impacting health care.

Theme	Perspectives of breast cancer patients
Delayed screening mammogram	*I suppose it might have benefited me if I had my mammogram last year. And you know, when I went in to get it, they said, oh, nobody got it [a mammogram] last year*. *I delayed my mammogram but that was a personal choice. The pandemic didn't limit my access, but my personal choice changed because of COVID. You know, like it was my choice not to go get my mammogram when I should have, just because I didn't want to be exposed*.
Delayed medical appointments and treatment	*I cancel appointments frequently because there's a lot of COVID. I don't want to be around, you know, I just don't want that extra visit to some office*. *There was a lot [delayed appointments]. I'm not going to the dentist or to the doctor just for a little cold or something like that. I wouldn't go. I just wouldn't go out*.

### 3.4 Perspectives on challenges during the COVID‐19 pandemic

Across interviews, several breast cancer patients noted how the COVID‐19 pandemic had created challenges for them. Interviewees were particularly focused on the feelings of anxiety and isolation they experienced after their diagnosis and during their treatment. Detailed examples of these challenges are described next, with additional representative quotations presented in Table 4.

**TABLE 4:** Perspectives on challenges during the COVID‐19 pandemic.

Theme	Perspectives of breast cancer patients
Anxiety about getting sick	*I am [concerned] because we have friends that have not been vaccinated and we have like, repair people that come here that haven't been vaccinated. And I have three friends that were vaccinated and got COVID. So, I really don't want to get it. I am concerned*. *Once I was finishing with chemo and before surgery, I started freaking out probably about getting COVID. Mainly because I had the pneumonia and I saw my chest x‐ray, and I saw what my lungs looks like. So, thinking about getting COVID on top of pneumonia was pretty scary*. *I've never really been an anxious person in my life. Cancer brought on a little anxiety but when you've got like your life when you have pneumonia, cancer, just got through chemo, your immune system's down and you're like afraid of getting COVID*.
Feeling isolated	*You have this big diagnosis. Maybe you're not getting like the family support, like, I haven't seen most of my family, like I said, other than my kids and my husband. You know, I normally would. They're a very loving family so they would have been here supporting me*. *I miss my friends. I miss my family. My sister and I are very close. I've seen her once in the last two years, you know, that sort of thing is very difficult*. *I'm a very contact person, personal, up close, in front, you know. I've always been that person, touchy feely. And I had to take and change that dynamic in my life because you know it's no more hugging. It's changed my life. And the cancer came along too. It's really made it, you know, you got to really be a person that you know, I can't stand out. I have to, you know, retreat, and come back, and you know, kind of be in this little bubble type thing*.

Fear of COVID‐19 infection was highlighted as a source of anxiety for breast cancer patients. One interviewee shared, “*[I was] scared to go out, and being scared to catch it, and being scared of everything. I have anxiety and panic attacks and it does a number on them. That's one of the reasons I retired because of it. Because it took a hold of that anxiety. And I just didn't want to leave out. I didn't want to go out and around anybody*.” Another interviewee similarly explained, “*After I got diagnosed and I started chemo and my all my counts went down, I've been terrified, honestly. And then for it [COVID‐19 infection] to happen to me. I was in the hospital for a while*.”

Breast cancer patients also described feelings of isolation associated with the COVID‐19 pandemic. One interviewee reflected, “*Just being isolated. I'm a very social person and to not be around people, you sit at home, and you get in your head a lot. I feel like a better, happier person when I'm able to go out and be around people. And when there's a pandemic and you're not allowed, or you can and you'll be afraid, it just makes it harder. Especially with being diagnosed and being treated now. I don't go anywhere, so, it's even worse*.” Another breast cancer patient shared, “*All of my treatment was under pandemic time, so I wasn't even, you know, allowed to have anyone with me or anything like that. From the standpoint of getting the treatments and going through all of the procedures alone. So, that was kind of nerve‐racking. Would have been nice to have the support system there with you*.”

### 3.5 Perspectives on positive outcomes during the COVID‐19 pandemic

Interestingly, several breast cancer patients described unexpected positive aspects of the pandemic in addition to sharing their perspectives about negative impacts and the challenges they had experienced. For example, having the ability to continue working because of the option to work from home was highlighted as a positive. One interviewee reflected, “*I will say, and this is kind of a weird positive in this case, but I'm kind of glad the pandemic happened when I was diagnosed with breast cancer because I was actually able to stay at home and work more than I would have been able to physically work had I had to still go into the office every day. I would not have been able to maintain a work schedule as well as I was able to because I could, my commute was from one room to the next*.” Regarding family, one interviewee commented, “*I think the [pandemic] made us all realize how precious family is*.” Another interviewee shared, “*I love the fact that it gave me new tools to deal with stressful situations from a different perspective. And I also was able to teach those tools to my kids, potentially for stressful situations that they'll encounter down the road*.” Another described how the pandemic had created a new perspective on life: “*This is just a silver lining in I do believe that this COVID is here to wake up people, you know. Like I said, the effect it has on me, I am more heartfelt with people now because you just never know when you can get a devastation. Yes, the compassion comes out, you know, the helping and loving your neighbor type thing*.”

## 4 DISCUSSION

Our study provides insight about the unique experiences of breast cancer patients during the COVID‐19 pandemic including perspectives surrounding decision making about vaccination, the impact of the pandemic on undergoing cancer treatment, delays in seeking or receiving health care, personal challenges experienced during the pandemic, and unexpected positive aspects of the pandemic. Our findings also underscore the need to encourage breast cancer screening activities and highlight some of the unanticipated positives that these women identified during the pandemic. These results also suggest the need to provide support for this patient population including offering information that supports COVID‐19 vaccination, considering patients' needs for social and mental health support throughout their treatment, and ensuring ongoing access to safe and timely health care.

Our findings supplement the results of other studies that have examined additional aspects of the experiences of breast cancer patients during the COVID‐19 pandemic. For example, qualitative studies have examined patients' attitudes about adhering to mammogram appointments,^23^ opinions about delays in breast cancer screening,^24^ and perceptions related to the diagnosis of breast cancer during the early waves of the pandemic, prior to the availability of COVID‐19 vaccines.^25^ Interviews with patients with metastatic breast cancer have also revealed perspectives about the use of telehealth for their cancer care and the use of virtual communication to participate in support groups,^26^ and interviews with breast cancer survivors have demonstrated mechanisms for coping with the challenges posed by the pandemic.^19^, ^20^, ^27^

Our study findings add to this breadth of knowledge by providing perspectives from patients in active treatment at a time in the pandemic during which COVID‐19 variants were contributing to infection surges and when vaccines were widely available. Our interviews therefore shed light on breast cancer patients' perspectives about COVID‐19 vaccination that have not been commonly reported in other qualitative studies of this patient population. The majority of the patients we interviewed had received a COVID‐19 vaccine and believed that vaccination was important to protect themselves, their families, and their communities. However, some participants had declined to be vaccinated and expressed concerns that are shared with members of the general public, including concerns about the safety of the vaccine.^28^ Interestingly, some participants also stated reasons they refused or delayed vaccination such as because they were already feeling unwell and did not want to experience potential side‐effects of vaccination. Others noted they did not want to be vaccinated prior to receiving their cancer therapy. These findings are aligned with the results of other studies that have investigated fear of COVID‐19 vaccination among cancer patients, including those with breast cancer.^29^ As COVID‐19 vaccines are important to protect cancer patients, since they are more susceptible to COVID‐19 infection and are at higher risk to experience severe disease caused by the virus,^30^ it is critical to continue efforts to promote vaccination in this population. Our findings suggest the opportunity to create tailored messages that address the specific questions breast cancer patients might have when making decisions about COVID‐19 vaccination.^31^, ^32^

Our findings also illuminate the concerning impact of COVID‐19 on the health care services received by breast cancer patients such as the influence of the pandemic on patients' decisions to access care. These findings align with evidence showing the pandemic has contributed to both decrease in breast cancer screening for individuals prior to their breast cancer diagnosis,^33^ and decrease in contact with health care providers for patients who were already diagnosed with breast cancer.^34^ While our study population consisted of patients who were undergoing active treatment, and therefore had already made decisions about their therapy, other studies have shown how the pandemic has impacted treatment decision making for breast cancer patients at other stages in their cancer journeys.^35^, ^36^ For example, fear of COVID‐19 infection may have led some patients to refuse treatment during the pandemic.^37^ Such delays in health care present risks both for increased anxiety^38^ as well as adverse clinical outcomes, although these impacts remain to be determined.^38^, ^39^ Recognizing organizational barriers and personal reasons for potential deficits in preventive and/or therapeutic care is critical to developing strategies that promote timely use of health care in this patient population, such as the use of telemedicine rather than in‐person visits when that is deemed appropriate.^40^, ^41^

Our study also revealed challenges experienced by breast cancer patients undergoing curative‐intent treatment during the pandemic, including feelings of anxiety and isolation that added to the stress of being diagnosed and treated for breast cancer. Our findings have been echoed in other studies that have demonstrated the negative effects of the pandemic on breast cancer patients' psychological well‐being through questionnaires evaluating insomnia, anxiety, depression, fear of cancer recurrence, and COVID‐19 stressors.^42^, ^43^, ^44^ Developing instruments to assess and monitor stress and impacts caused by concerns related to COVID‐19 may be an important step in identifying breast cancer patients who need additional support to face these challenges.^45^

Finally, many interview participants noted unexpected positive aspects of undergoing breast cancer treatment during the pandemic. These positive aspects included the ability to continue working safely from home and enhanced appreciation for and connections with family. Identifying these types of positives may help individuals facing difficult situations to build their psychological resilience,^46^ an important factor in helping breast cancer patients cope with the anxiety of diagnosis, treatment, and survivorship.^47^ Interestingly, unexpected positives have also been described by breast cancer health care providers who recognize that new care delivery methods, such as telemedicine, that were expanded and/or adapted in response to the COVID‐19 pandemic may benefit patients in the long run.^48^, ^49^

### 4.1 Limitations

Several limitations should be considered when interpreting the results of this study. First, our patient population is limited to a single clinical site and therefore patient experiences at different health care organizations may differ. Second, selection bias may exist in the recruitment of our patient participants, in that patients with heightened concerns about COVID‐19 may have been more likely to participate. Nonetheless, the diversity of views represented, for example the inclusion of both vaccinated and unvaccinated participants as well as across age ranges, gives us confidence about the salience of the findings we report. Third, while our study focused on patients currently undergoing breast cancer treatment, these findings may translate to breast cancer survivors as prior studies have also shown the pandemic's negative effects on emotional well‐being, loneliness, and willingness to seek health care for this patient population.^50^, ^51^ Additional work is needed to further understand the unique pandemic experiences of breast cancer survivors and identify their particular needs.

## 5 CONCLUSION

This work highlights the needs and concerns of breast cancer patients undergoing neoadjuvant chemotherapy as they have faced the challenges of navigating breast cancer treatment during a critical period in the COVID‐19 pandemic that spans delta and omicron variant waves and during which there was widespread availability of COVID‐19 vaccines. Understanding these perspectives will help providers and organizations consider adaptation of approaches to support breast cancer patients in the future, both as the COVID‐19 pandemic continues to evolve, and in preparation for other potential public health emergencies that impact this high‐risk patient population. Addressing the challenges faced by this patient population can serve to improve the health, well‐being, and safety of these vulnerable patients.

## AUTHOR CONTRIBUTIONS

**Alice A. Gaughan:** Conceptualization (equal); data curation (equal); formal analysis (equal); funding acquisition (equal); investigation (equal); project administration (equal); visualization (equal); writing – original draft (equal); writing – review and editing (equal). **Sarah R. MacEwan:** Conceptualization (equal); formal analysis (equal); investigation (equal); visualization (equal); writing – original draft (equal); writing – review and editing (equal). **Laura J. Rush:** Conceptualization (equal); formal analysis (equal); investigation (equal); visualization (equal); writing – original draft (equal); writing – review and editing (equal). **Margaret E. Gatti‐Mays:** Conceptualization (equal); resources (equal); writing – original draft (equal); writing – review and editing (equal). **Ashley C. Pariser:** Conceptualization (equal); resources (equal); writing – original draft (equal); writing – review and editing (equal). **Ann Scheck McAlearney:** Conceptualization (lead); formal analysis (equal); funding acquisition (lead); investigation (lead); resources (lead); supervision (equal); visualization (equal); writing – original draft (equal); writing – review and editing (equal).

## FUNDING INFORMATION

This study was supported by a grant (# U54 CA260582) from the National Cancer Institute (NCI), but the NCI was not involved in the data collection, analysis, or the decision to present these findings.

## CONFLICT OF INTEREST STATEMENT

The authors declare that the research was conducted in the absence of any commercial or financial relationships that could be construed as a potential conflict of interest.

## ETHICS STATEMENT

This study involving human participants was approved by OSU's Institutional Review Board.

## Data Availability

Data from this study will not be made publicly available due to concerns for participant privacy.

## APPENDIX A INTERVIEW GUIDE

### A.1

**Section 1: Introduction and background**

- To start, for our records, could you describe your type of cancer and the treatment(s) you are receiving?
- In which county do you live?

**Section 2: Perspectives about COVID‐19 vaccination**

- Did you get a COVID‐19 vaccine?
  - If yes,
    ▪ What made you decide to get the vaccine?
    ▪ Did you have concerns about the vaccine (e.g., safety, side effects, efficacy in persons with depressed immune systems)?
    ▪ Have you been tested for antibodies?
  - If no,
    ▪ What made you decide not to get the vaccine?
    ▪ Would you be more willing to get the vaccine if it receives final FDA approval rather than the current emergency use approval?
- Has your healthcare team discussed COVID and the COVID vaccine with you? What types of things have you discussed with them? (e.g., safety, side effects, efficacy in persons with depressed immune systems)
- Have you had any conversations with your friends and family about COVID‐19 vaccines?
  - If yes, what have these conversations been about?
- If a booster dose of the COVID‐19 vaccine becomes available, would you be willing to get it?

**Section 3: Perspectives on COVID‐19 risk**

- Are you currently concerned about your risk of exposure to COVID‐19 from friends and/or family?
  - If yes,
    ▪ Can you describe your concerns?
    ▪ Have you had any conversations with people about these concerns? (What have those conversations been about?)
    ▪ What have you done about these concerns? (e.g., continue to wear a mask)
- Do you have particular concerns about entering healthcare facilities?
  - Have these changed over time?
- What precautions are you currently taking to protect yourself from COVID‐19?
  - Have these changed over time?
  - Have these changed after you were vaccinated? (If yes to vaccination)
  - Have these changed in response to the emergence of COVID‐19 variants?
- Has the emergence of the delta variant impacted your behaviors?
  - If yes, has this impacted
    ▪ Your likelihood of getting a vaccine/desire to get a booster dose?
    ▪ Your use of PPE?
    ▪ Your decisions about gathering with other people?
    ▪ Your decisions about where/whether to travel?

**Section 4: Perspectives on COVID‐19 viral testing**

- Have you ever had a viral test for COVID‐19 (e.g., the nasal swab—this tells you if you are actively infected with the coronavirus)?
  ***If Yes*** :
- Where did you get tested?
  - Were there options about where to get tested or what type of test to receive?
  - How did you hear about testing options?
- Why did you decide to get tested? (e.g., close contact tested positive)
- If close contact was positive, what did you do?
  - Did you receive any guidance about what to do with respect to interactions? Contact tracing?
  - What changes did you make to your interactions?
  - Did you contact any more of your own contacts?
  - Once your close contact was out of isolation, what happened next with respect to your isolation and/or interactions?
- What did you think about the test and the testing process?
- Did you experience any barriers to getting tested? (e.g., information, need to have symptoms, confusion about type of test)
  - What were they?
- How were your test results communicated to you? (e.g., through a portal—have to log in or not?)
  - What information was included with your results?
  - Was there anything about the test results that you did not understand?
  - Was there any other information you wish you had received after getting tested? (e.g., quarantining guidelines, more clarity about what to do next)
- Did you receive any follow‐up after you got tested (e.g., did a health care provider or the testing site reach out to you)?
- Did you have to report your test results or exposure or both somewhere?
- If you got re‐tested, was the process different?
  - Was the experience different?

**Section 5: Perspectives on COVID‐19 serological testing**

- Have you ever had a serological test for COVID‐19?
  ***If Yes*** :
- Why did you get the test?
- Where were you tested?
- How were the test results communicated to you? (e.g., through a portal—have to log in or not?)
  - What information was included with your results?
- Do you understand what to do with your results?
- Did the test results change your behavior? (e.g., social distancing, mask wearing)
- Did you experience any barriers or challenges to getting tested? (e.g., cost)
- Is there anything that would have made getting tested easier? (e.g., cost)
- Did you receive any follow‐up after you got tested?
  - Can you please explain what that process looked like?
    **OR**
  - If you needed follow‐up regarding your test results, where would you go?
  - Is there anything you wish was done differently during follow‐up?

**Section 6: Confusion around COVID‐19 guidance**

- Where have you received information about COVID‐19 exposure and transmission? (e.g., your employer, your doctor)
  - How was that information provided to you?
  - What type of guidance have you received?
- Are you confused at all about guidance around mask wearing, contact tracing, etc.?
- Have you had to contract trace any of your close contacts?
  - What was your experience with that?
- Are there other areas where you wish there had been better guidance around COVID‐19? (e.g., how to quarantine, the need to contact trace)
- Have you received information about the downstream health consequences of COVID‐19?
  - How was that information provided to you?

**Section 7: Perspectives on impact of COVID‐19**

- How has the COVID‐19 pandemic affected your access to or experiences with healthcare?
- Did you delay any medical appointments, treatments, or procedures (related to your cancer treatment or other healthcare needs) due to the COVID‐19 pandemic?
- Do you have any concerns about your cancer care during the COVID‐19 pandemic?
- What are the greatest challenges that you see now with this pandemic? (e.g., fatigue, vaccine, supplies, economic impact)
- What are you concerned about looking farther ahead?
- Can you describe the impact of this pandemic on yourself?
- How do you think the pandemic has impacted the mental health of you or your family members/household contacts?

**INTERVIEW CLOSURE AND FOLLOW‐UP**

- Is there anything else we should know or that you would like to tell us regarding these topics?

**Thank you so much for your time and participation. Your comments were extremely helpful.**
